# Role of wearable devices in cardiac telerehabilitation: A scoping review

**DOI:** 10.1371/journal.pone.0285801

**Published:** 2023-05-31

**Authors:** Alexis K. Jones, Crystal Lihong Yan, Beatriz P. Rivera Rodriquez, Sukhpreet Kaur, Sharon Andrade-Bucknor

**Affiliations:** 1 University of Miami Miller School of Medicine, Miami, FL, United States of America; 2 Department of Medicine, University of Miami/Jackson Memorial Hospital, Miami, FL, United States of America; 3 Department of Medicine, Division of Cardiovascular Disease, University of Miami/Jackson Memorial Hospital, Miami, FL, United States of America; University of Campania Luigi Vanvitelli: Universita degli Studi della Campania Luigi Vanvitelli, ITALY

## Abstract

**Background:**

Cardiac rehabilitation (CR) is an evidence-based comprehensive program that includes exercise training, health education, physical activity promotion, and extensive counseling for the management of cardiovascular risk factors. Wearable devices monitor certain physiological functions, providing biometric data such as heart rate, movement, sleep, ECG analysis, blood pressure, energy expenditure, and numerous other parameters. Recent evidence supports wearable devices as a likely relevant component in cardiovascular risk assessment and disease prevention. The purpose of this scoping review is to better understand the role of wearable devices in home-based CR (HBCR) and to characterize the evidence regarding the incorporation of wearable devices in HBCR programs and cardiovascular outcomes.

**Methods & findings:**

We created a search strategy for multiple databases, including PubMed, Embase (Elsevier), CINAHL (Ebsco), Cochrane CENTRAL (Wiley), and Scopus (Elsevier). Studies were included if the patients were eligible for CR per Medicare guidelines and >18 years of age and if some type of wearable device was utilized during HBCR. Our search yielded 57 studies meeting all criteria. The studies were classified into 4 groups: patients with coronary heart disease (CHD) without heart failure (HF); patients with HF; patients with heart valve repair or replacement; and patients with exposure to center-based CR. In three groups, there was an upward trend toward improvement in quality of life (QOL) and peak VO2, less sedentary time, and an increase in daily step count in the intervention groups compared to control groups.

**Conclusions:**

HBCR using wearable devices can be a comparable alternative or adjunct to center-based CR for patients with CHD and HF. More studies are needed to draw conclusions about the comparability of HBCR to center-based CR in patients with heart valve repair or replacement.

## Introduction

Cardiac rehabilitation (CR) is a personalized, evidence-based, and supervised comprehensive program that includes exercise training, health education, physical activity promotion, and extensive counseling for the management of cardiovascular risk factors [1]. Aside from being a relevant component for secondary prevention, it has been shown to reduce morbidity and mortality in certain cardiac conditions [2, 3]. The American Heart Association and American College of Cardiology guidelines assign a Class I indication for referral to CR for patients after an acute myocardial infarction, coronary revascularization (percutaneous coronary intervention (PCI) or coronary artery bypass graft (CABG) surgery), chronic stable angina, and heart failure with reduced ejection fraction (HFrEF) [2, 3]. Referral after valve surgery and cardiac transplantation has also been shown to be beneficial [2, 3]. CR is covered for all the aforementioned indications by Medicare and Medicaid services, and it is even included in the consensus core set for cardiovascular performance measures as an area of future development [4]. Despite the strong recommendation for the referral of these patient groups, access to CR remains poor [1, 3]. The recent COVID-19 pandemic has further contributed to the reduction in access to CR programs [1]. Given these obstacles, research around telerehabilitation programs has increased over the past few years.

As technology advances, it permeates all aspects of our lives and plays an increasingly important role in medicine. One such aspect is wearable devices or technology. Wearable devices refer to small electronic and mobile devices or computers with communication capabilities incorporated into gadgets, clothes, or even accessories, that can be worn on the human body. This technology has the ability to monitor certain physiological functions, providing biometric data such as heart rate, movement, sleep, ECG analysis, blood pressure, energy expenditure, and numerous other parameters [5]. As such, studies have examined the use of wearable devices in movement disorders such as multiple sclerosis [6, 7]. Furthermore, recent evidence supports wearable devices as a likely relevant component in cardiovascular risk assessment and disease prevention [8]. CR has many barriers, including low attendance because of the inconveniences and costs associated with attending in-person sessions [8, 9]; however, the use of these devices has the potential to overcome these barriers and allow both affordable and reliable care from the comfort of the patient’s own home [3, 10]. Despite these promising initial results, there is still a need to further examine the role of wearable devices on a larger scale and to understand how their use can affect cardiovascular outcomes in patients who undergo CR.

The purpose of this scoping review is to better understand the role of wearable devices in home-based CR (HBCR) and to characterize the evidence regarding the incorporation of wearable devices in HBCR programs and cardiovascular outcomes. Because this technology is still novel and under development, it is relevant to identify its uses and limitations, so it can be more effectively integrated into CR programs in the future. The integration of wearable technology into CR has the potential to create more sustainable CR models that would narrow the gap in accessibility in underrepresented groups and low to mid-income areas.

## Methods

We conducted a scoping review and worked closely with a librarian to ensure that we were following standard guidelines. A study protocol was published on OSF (https://osf.io/yvq2j/). This review aims to give researchers an overview of the role of wearable devices in HBCR. We chose our inclusion criteria based on Medicare guidelines and using a broad definition of a wearable device to ensure that we could provide a broad overview of this topic.

### Inclusion criteria

#### Types of participants

Studies were included if the patients were eligible for CR as per Medicare guidelines regardless of sex or ethnicity. Patients must have had at least one of the following conditions: acute myocardial infarction in the last 12 months, coronary artery bypass surgery, current stable angina, heart valve repair or replacement, coronary angioplasty or coronary artery stent, heart or a heart-lung transplant, and stable chronic heart failure. Studies of patients younger than 18 years old were excluded from the review.

#### Concept

The core concept of this scoping review was to assess the use of wearable devices. Any study that utilized some type of wearable device during HBCR was eligible for inclusion. Wearable device was defined as any device that can be worn on any part of the body that has the capability of recording biometric data used as parameters for monitoring CR. Many wearable devices are associated with smartphone or web-based applications, and studies that include the usage of both the device and the application were eligible for inclusion. However, studies that solely use an application without a device were not eligible.

#### Context

The context element of this scoping review is limited to CR that is home-based or at least with a home-based component. CR taking place in only a medical facility, either a rehabilitation center or inpatient, were not eligible either.

#### Types of evidence sources

Primary research studies were included as the source of the information for this scoping review. Review articles such as systematic reviews and meta-analyses were reviewed for primary research studies but not included as a source of information in their entirety. Case reports, letters, abstracts, and opinion articles were excluded. We excluded any articles outside of English and Spanish languages where the translation was not available. Primary research studies were excluded if no results were reported.

### Search strategy

**Fig 1:**
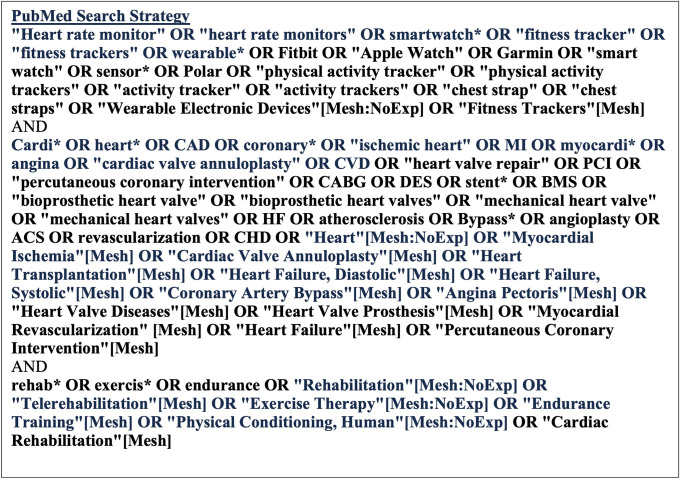
PubMed search strategy.

Our search strategy consisted of three steps and searched health and science and multidisciplinary databases for published studies and reviews. The first step involved searching PubMed with a more limited strategy to identify additional keywords. Additionally, the results from these searches were analyzed using the Yale MeSH Analyzer (http://mesh.med.yale.edu). Next, a comprehensive search strategy was written for PubMed, peer-reviewed by a librarian, and adapted for other databases including Embase (Elsevier), CINAHL (Ebsco), Cochrane CENTRAL (Wiley), and Scopus (Elsevier). Fig 1 shows the PubMed search strategy. This search was modified and rerun as needed to ensure that all data sources were included. The final searches for each data base were run in March 2023.

The reference lists of studies were examined and extracted from the main databases by two authors. The extracted citations of the studies from the initial search were placed on Endnote and then uploaded to Covidence (https://app.covidence.org/) by one reviewer. Covidence eliminated duplicates. The initial search was only limited by language, only including articles either in Spanish or English or with an available translation.

### Source of evidence selection

Selection of the titles and abstracts from the initial search was performed independently by five reviewers. For inclusion or exclusion of an article, two reviewers had to agree. If there were discrepancies in the study selection, a third reviewer made the final decision. After the initial evidence selection was finalized, the full text of all potentially eligible studies was read by four authors independently. The team met to discuss discrepancies and disagreements of the articles selected and reached a consensus by either the team or by a third party. Review articles that met inclusion criteria were not included as a whole but rather the individual studies from each review article were evaluated for inclusion or exclusion.

### Data extraction

Data was extracted by four reviewers independently using a form that had been tested by the team before their use. The extracted data was then confirmed by a second reviewer.

### Data items

Study and participant characteristic variables were collected and included the country the study was conducted, number of study participants, number of study participants who did not complete the study, study population, study design, CR phase, and participant age and sex. Furthermore, the type of wearable device, intervention description, intervention duration, control group description, study outcomes, and study results were collected.

## Results

**Fig 2:**
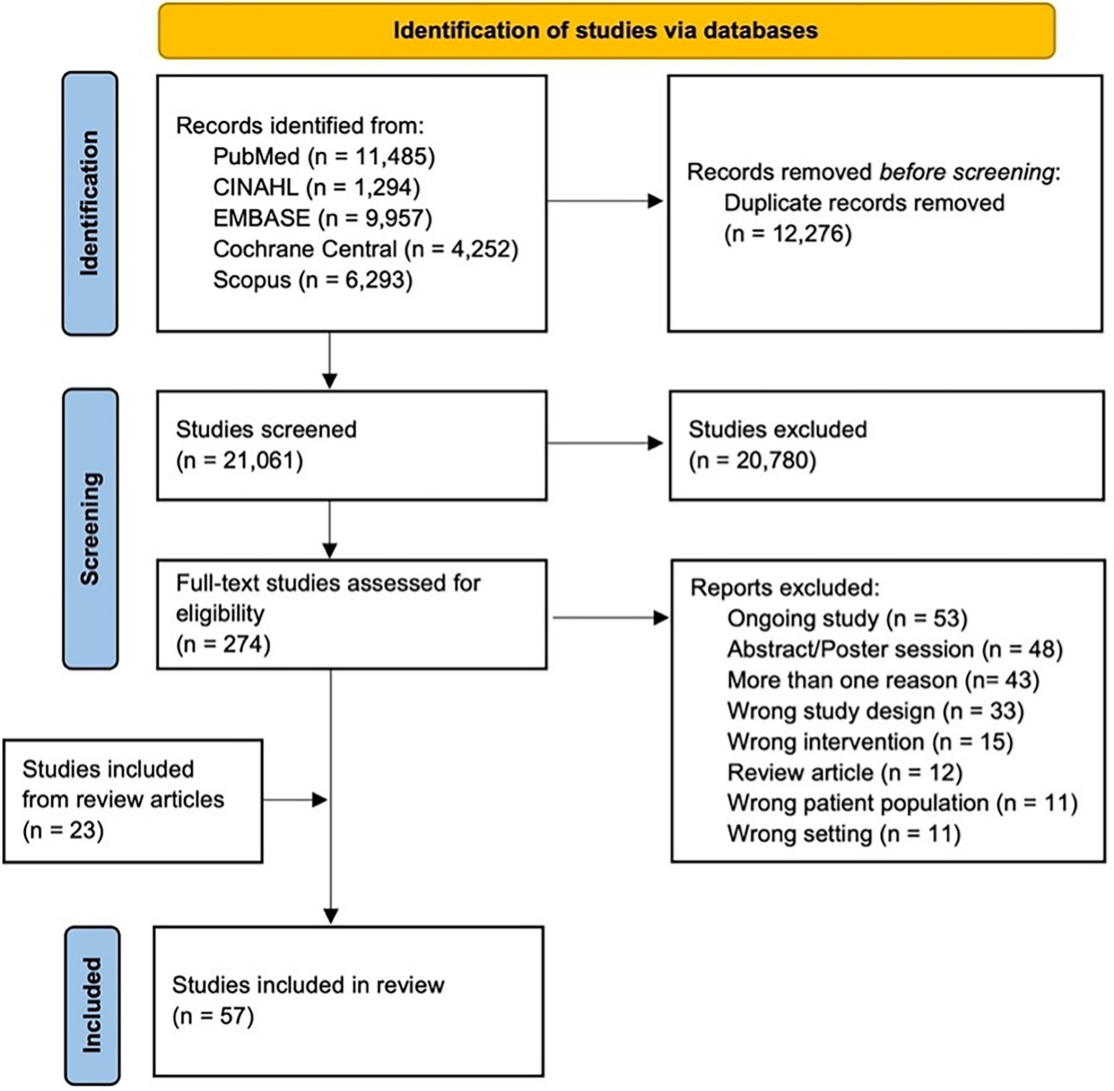
PRISMA flow diagram.

Fig 2 shows a PRISMA diagram [11] that demonstrates our study selection process. We searched five databases (PubMed, CINAHL, EMBASE, Cochrane Central, and SCOPUS). Covidence removed 12,276 duplicates leaving us with 21,061 articles to screen. We then screened titles and abstracts for relevance. In this initial screening process, 20,780 studies were removed. The full texts for the remaining 274 articles were reviewed to determine if they met eligibility criteria. At this point, 23 individual articles that met eligibility criteria from review articles were added. A total of 57 articles met our criteria.

We found 57 studies from 2003 to 2022, of which there were 44 randomized controlled trials, 9 cohort studies, and 2 non-randomized trials. There were four main group classifications:

- Patients with CHD without HF (n = 27)
- Patients with HF (n = 15) and
- Patients with exposure to center-based CR (n = 14)
- Patients with heart valve repair or replacement (n = 1)

Table 1 summarizes the study and participant characteristics of each study. The most studies were conducted in the United States at 8 studies [12–19], followed by 6 studies in Belgium [20–25]; 4 in Canada [17, 26–28], the Czech Republic [29–32], Japan [33–36], the Netherlands [37–40], and Poland [41–44]; 3 in China [45–47], and Spain [48–50]; 2 in Australia [51, 52], France [17, 53], Germany [49, 50], Iran [54, 55], Italy [56, 57], and New Zealand [58, 59]; and 1 in Denmark [60], Finland [61], Israel [62], Kenya [63], Korea [64], and Portugal [65]. Three studies were conducted across 3 different countries [17, 49, 50]. Of the 57 studies, 39 were conducted in phase 2 CR only, 16 in phase 3 CR only, and 2 in both CR phases 2 and 3 [13, 61]. The number of participants ranged from 5 [28] to 2331 [17]. The number of participants who did not complete the study ranged from 0 [26, 44, 52, 57, 66] to 150 [58] participants, translating to an incompletion rate of 0% to 92.6%. Three studies did not report a dropout rate [37, 19, 62]. The mean age of the study participants ranged from 54.17 [47]– 74 [36] years in intervention group and from 54.83 [47]– 70 [19] in the control group. Mean age was not reported in 3 studies [37, 46, 56], and 1 study did not report age for the control group [59]. Gender recruitment was significantly imbalanced. Most studies included >70% males compared to females. Three studies did not report gender breakdown [37, 56, 65] and 2 studies did not report gender breakdown for the control group [54, 65].

**Table 1:** Study and participant characteristics.

Author (year)	Country	No. participants	No. incomplete	Study design	CR phase	Age in years Mean (SD)	Male %
Ades (2000)	United States	145	12	Non-randomized controlled trial	Phase 2	I: 56 (9) C: 58 (12)	I: 76% C: 90%
Avila (2018)	Belgium	90	6	RCT	Phase 3	I: 58.6 (13) C: 61.1 (7.7)	I: 87% C: 90%
Avila (2020)	Belgium	90	10	RCT	Phase 3	I: 62.2 (7.1) C: 88%	I: 63.7 (7.4) C: 92%
Batalik (2020)	Czech Republic	56	5	RCT	Phase 2	I: 56.5 (6.9) C: 57.7 (7.6)	In: 80% C: 85%
Batalik (2021)	Czech Republic	56	5	RCT	Phase 2	I: 56.1 (6.8) C: 57.2 (7.5)	I: 79% C: 84%
Batalik (2021)	Czech Republic	56	12	RCT	Phase 2	I: 56.1 (6.8) C: 57.1 (7.9)	I: 78% C: 86%
Batalik (2021)	Czech Republic	19	3	Prospective cohort	Phase 2	60.4 (9.6)	74%
Bravo-Escobar (2017)	Spain	28	1	RCT	Phase 2	I: 56.5 (6.01) C: 55.64 (11.35)	I: 100% C: 100%
Brocki (2022)	Denmark	13	8	Prospective cohort	Phase 2	82 (N/A)	60%
Brouwers (2021)	Netherlands	300	N/A	RCT	Phase 2	I: N/A C: N/A	I: N/A C: N/A
Butler (2009)	Australia	110	20	RCT	Phase 3	I: 63 (10.4) C: 64.5 (11.2)	I: 69.1% C: 81.8%
Chokshi (2018)	United States	105	1	RCT	Phase 2 and 3	I: 60.0 (9.5) C: 59.1 (11.5)	I: 72% C: 67.3%
Cupples (2013)	United Kingdom	45	3	RCT	Phase 3	I: 61.6 (11.3) C: 59.2 (8.9)	I: 84.2% C: 96.2%
Dehghani (2019)	Iran	55	5	RCT	Phase 2	I: 51.4 (8.0) C: 51.1 (7.9)	I: 50% C: 50%
Dehghani (2022)	Iran	95	15	RCT	Phase 2	I: 51.4 (8.0) C: 51.1 (7.9)	I: N/A C: N/A
Devi (2014)	United Kingdom	95	22	RCT	Phase 2	I: 66.27 (8.35) C: 66.20 (10.06)	I: 71% C: 78%
Ding (2021)	United States	20	2	Prospective cohort	Phase 2	58 (7)	67%
Dolansky (2010)	United States	273	25	Prospective cohort	Phase 3	62.4 (11.1)	61.7%
Duscha (2018)	United States	25	7	RCT	Phase 3	I: 59.9 (8.1) C: 66.5 (7.2)	I: 81.2% C: 66.7%
Fang (2019)	China	80	13	RCT	Phase 2	I: 60.24 (9.35) C: 61.41 (10.17)	I: 63.6% C: 61.8%
Frederix (2015)	Belgium	140	14	RCT	Phase 2	I: 61 (9) C: 61 (8)	I: 86% C: 79%
Frederix (2015)	Belgium	80	14	RCT	Phase 3	I: 58 (9) C: 63 (10)	I: 81% C: 85%
Frederix (2016)	Belgium	140	14	RCT	Phase 2	I: 61 (9) C: 61 (8)	I: 86% C: 79%
Frederix (2017)	Belgium	140	14	RCT	Phase 2	I: 61 (9) C: 61 (8)	I: 84% C: 80%
Frith (2021)	United Kingdom	46	0	Prospective cohort	Phase 3	60.4 (3.3)	78.3%
Guiraud (2012)	France	29	13	RCT	Phase 2	I: 54.5 (12.6) C: 62.9 (10.7)	I: 89% C: 70%
Hakala (2021)	Finland	59	6	RCT	Phase 2 and 3	I: 59.7 (6.0) C: 59.2 (6.1)	I: 76% C: 87%
Hannan (2021)	Australia	20	0	Within-subject controlled trial	Phase 3	56 (15.5)	83%
Izawa (2005)	Japan	50	5	RCT	Phase 3	I: 63.9 (9.7) C: 64.5 (10.1)	I: 87.5% C: 81.0%
Jafri (2022)	United States	269	N/A	Retrospective cohort	Phase 2	I: 73 (9) C: 70 (11)	I: 98% C: 99%
Kikuchi (2021)	Japan	11	1	Prospective cohort	Phase 2	76 (7)	60%
Kraal (2014)	Netherlands	55	5	RCT	Phase 2	I: 60.6 (7.5) C: 56.1 (8.7)	I: 88% C: 84%
Kraal (2017)	Netherlands	90	12	RCT	Phase 2	I: 60.5 (8.8) C: 57.7 (8.7)	I: 89% C: 89%
Lear (2015)	Canada	78	0	RCT	Phase 2	I: 61.7 C: 58.4	I: 90% C: 80%
Lee (2013)	Korea	60	55	RCT	Phase 2	I: 54.3 (8.9) C: 57.8 (7.5)	I: 85% C: 76%
Maddison (2019)	New Zealand	162	150	RCT	Phase 2	I: 61.0 (13.2) Cl: 61.5 (12.2)	I: 84.2% C: 87.5%
Marchionni (2003)	Italy	270	38	RCT	Phase 2	I: N/A C: N/A	I: N/A C: N/A
Nabutovsky (2022)	Israel	306	N/A	Retrospective cohort	Phase 2	57.6 (10.6)	81.5%
Nagatomi (2022)	Japan	30	0	RCT	Phase 2	I: 59.8 (10.0) C: 67.7 (8.9)	I: 60% C: 47%
Ngeno (2022)	Kenya	100	12	Non-randomized controlled trial	Phase 2	I: 44 (16) C: 54 (16)	I: 10% C: 39%
O’Connor (2009)	United States, Canada, France	2331	26	RCT	Phase 2	I: 59.2 C: 59.3	I: 70.1% C: 73.2%
Park (2021)	United States	60	9	RCT	Phase 3	I: 66.7 (8.6) C: 66.8 (8.7)	I: 77% C: 80%
Peng (2018)	China	98	15	RCT	Phase 2	I: N/A C: N/A	I: 57.1% C: 61.2%
Piotrowicz (2010)	Poland	152	21	RCT	Phase 2	I: 56.4 (10.9) C: 60.5 (8.8)	I: 85% C: 95%
Piotrowicz (2015)	Poland	111	4	RCT	Phase 2	I: 54.4 (10.9) C: 62.1 (12.5)	I: 95% C: 97%
Piotrowicz (2020)	Poland	850	69	RCT	Phase 2	I: 62.6 (10.8) C: 62.2 (10.2)	I: 88.7% C: 88.5%
Prince (2018)	Canada	40	3	RCT	Phase 2	I: 62.4 (10.7) C: 61.5 (9.7)	I: 62% C: 59%
Rawstorn (2018)	New Zealand	162	28	RCT	Phase 2	I: 61.3 C: N/A	I: 85% C: N/A
Saitoh (2022)	Japan	11	0	RCT	Phase 3	I: 74 C: 68	I: 67% C: 80%
Salvi (2018)	Spain, Germany, United Kingdom	118	57	RCT	Phase 3	I: 60 C: 58	I: 91% C: 87%
Scalvini (2009)	Italy	47	0	Prospective cohort	Phase 2	64 (11)	87%
Skobel (2017)	Spain, Germany, United Kingdom	118	57	RCT	Phase 3	I: 60 C: 58	I: 91% C: 87%
Smolis-Bak (2015)	Poland	52	0	RCT	Phase 2	I: 60 (8.5) C: 65.1 (8.2)	I: 96.1% C: 84.6%
Snoek (2021)	Netherlands	122	4	RCT	Phase 3	I: 60 (8.4) C: 59 (10.7)	I: 82% C: 82%
Song (2020)	China	106	10	RCT	Phase 2	I: 54.17 (8.76) C: 54.83 (9.13)	I: 89.6% C: 83.3%
Tousignant (2019)	Canada	5	1	Prospective cohort	Phase 2	66.25	100%
Vieira (2017)	Portugal	46	13	RCT	Phase 3	I1: 55 (9) I2: 59 (11.3) C: 59 (5.8)	I: N/A C: N/A

Abbreviations: C control; CR cardiac rehabilitation; I intervention; RCT randomized controlled trial; SD standard deviation.

Table 2 summarizes the parameters and outcomes for each study. The common outcomes measured in these studies were cardiovascular (CV) risk factors, costs, daily steps, exercise capacity, physical activity (PA), psychological status, quality of life (QOL), and user experience. Psychological status was measured using various questionnaires for depression, anxiety, and self-efficacy such as the Hospital Anxiety and Depression Scale and General Self-Efficacy Scale. Similarly, QOL was measured using various questionnaires such as the SF-36 and HeartQol questionnaires. Nineteen studies mentioned risk factor outcomes with 7 reporting no difference between the two groups [20, 21, 27, 34, 40, 48, 50] and 2 reporting improved waist-hip circumference [58, 66]. The wearable devices used varied. Seventeen studies used heart rate monitors [15, 17, 20, 25, 26, 29–31, 37–40, 46, 47, 52, 56]. 10 used ECG monitors [12, 28, 34, 41–44, 48, 57, 64], 10 used pedometers [13, 18, 19, 33, 36, 51, 54, 55, 63, 67], 9 used accelerometers [21–24, 37, 53, 65, 66, 68], 7 used health watches [14, 16, 18, 35, 60–62], and 6 used other wearable sensors [27, 45, 49, 50, 58, 59].

**Table 2:** Study parameters and outcomes.

Author (year)	Wearable device	Study population	Intervention; duration	Control	Outcomes	Results
**Patients with coronary heart disease (without heart failure)**						
Ades (2000)	ECG leads connected to a transmitter unit	Had acute coronary event within 3 months	Home-based exercise program using an ergometer with real-time telephone feedback by a nurse during exercise + preventative education group calls; N/A	Center-based CR II	Exercise capacity, QOL	Both groups had significant improvements in peak VO2, peak workload, and QOL and there was no difference between groups.
Batalik (2020)	HR monitor (M430 Polar), wrist	Angina, MI, PCI, CABG	2 center-based training sessions, followed by a home-based exercise program with training data uploaded to a web application and telephone feedback by a PT 1x/week; 12 weeks	Center-based CR II	Exercise capacity, QOL	Both groups had significant improvement in peak VO2 and QOL with no difference between groups. Neither group had improvement in peak work load.
Batalik (2021)	HR monitor (M430 Polar), wrist	Angina, MI, PCI, CABG + low to moderate risk	2 center-based training sessions, followed by a home-based exercise program with training data uploaded to a web application and telephone feedback by a PT 1x/week; 12 weeks	Center-based CR II	Exercise capacity	There was no difference between groups for peak VO2, peak HR, or peak respiratory exchange ratio.
Batalik (2021)	HR monitor (M430 Polar), wrist	Angina, MI, PCI, CABG + low to moderate risk	2 center-based training sessions, followed by a home-based exercise program with training data uploaded to a web application and telephone feedback by a PT 1x/week; 12 weeks	Center-based CR II	Exercise capacity, QOL	The intervention group had significantly improved peak VO2 compared to the control group at 1 year follow up. There was no significant difference between groups for other measures of exercise capacity.
Batalik (2021)	HR monitor (M430 Polar), chest sensor	MI, PCI, CABG + low to moderate risk	Home-based exercise with telephone feedback by a PT 1x/2 weeks; 8 weeks	None	Exercise capacity	Participants significantly improved their 200m fast walk test time. There was no difference in max HR at the end of the test, resting HR, or rate of perceived exhaustion.
Bravo-Escobar (2017)	ECG monitor (NUUBO), vest	Stable ischemic CM + PCI/CABG + moderate risk	Hybrid exercise program with center-based CR 1x/week and home-based exercise at least 2x/week; N/A	Center-based CR II	CV risk factors, exercise capacity, QOL	Both groups had significant improvement in exercise time and METs during an exertion test with no difference between groups. QOL improvement occurred in the control group only. There were no significant changes in CV risk factors.
Brouwers (2021)	Accelerometer (ActiGraph), hip + HR monitor (Mio Alpha), wrist	Stable angina, ACS, PCI, CABG	6 center-based training sessions, followed by home-based exercise program with training data uploaded to a web application and video feedback by a PT 1x/week; N/A	Center-based CR II	PA, psychological status, QOL	Both groups had significant improvement in PA, QOL, depression, and patient empowerment with no difference between groups.
Chokshi (2018)	Pedometer (Misfit Shine), wrist	UA, NSTEMI, STEMI, PCI, CABG	Home-based exercise program with daily feedback and loss-framed financial incentive; N/A	Step count recorded without feedback	Daily steps	The intervention group had a significantly greater increase in mean daily steps from baseline during ramp-up, maintenance, and follow-up compared to the control group.
Dehghani (2022)	Pedometer (PA-S20), waist	MI, PCI	Home-based walking program with pedometer feedback; 8 weeks	Center-based CR without pedometer feedback	Exercise capacity	The intervention group had significantly improved METs, peak VO2, and total exercise times and distances compared to the control group.
Dehghani (2022)	Pedometer (PA-S20), waist	MI, PCI	Morning or evening home-based walking program with follow up calls from the research team every other day; 8 weeks	Usual care- counseling about routine physical activities	CV risk factors, exercise capacity	The intervention group had significant improvement in CV risk factors and peak VO2 compared to the control group. Furthermore, the evening intervention group had significant improvement over the morning intervention group.
Devi (2014)	Accelerometer (Sensewear Pro)	Stable angina	Internet-based education program to set individualized tailored lifestyle goals (exercise, diet, emotions, smoking); 6 weeks	Usual care- annual check for CV risk factor management	CV risk factors, daily steps, PA, psychological status, QOL	The intervention group had significantly improved daily step count, energy expenditure, duration of sedentary activity, duration of moderate activity, weight, self-efficacy, and emotional QOL compared to the control group. The control group had significantly reduced systolic BP compared to the intervention group. There were no between-group differences for body fat %, diastolic BP, diet, anxiety, and depression, among others.
Ding (2021)	Health watch (Phillips Healthcare)	MI + declined center-based CR	Home-based exercise program with telephone feedback 1x/week; 12 weeks	None	Daily steps, QOL	Participants’ daily step counts remained steady over the intervention period. QOL did not change significantly.
Fang (2019)	Belt strap with sensor	PCI + low risk	Home-based exercise program with telephone calls 1x/week and 2 home visits by a PT + educational booklet on CV risk factors; 6 weeks	Usual care- booklet and biweekly outpatient review	CV risk factors, exercise capacity, psychological status, QOL	Both groups had significantly improved 6-minute walk distance, anxiety/depression, nicotine dependence, and QOL; however, the intervention group had significantly greater improvement in 6-minute walk distance, nicotine dependence, and QOL compared to the control group.
Frederix (2015)	Accelerometer (Yorbody)	ACS + PCI/CABG	Center-based CR II, followed by a home-based exercise program with feedback; 18 weeks	Center-based CR II, then step count recorded without feedback	CV risk factors, exercise capacity, rehospitalization	The intervention group had significantly improved peak VO2 and a trend toward fewer rehospitalizations compared to the control group.
Kraal (2014)	HR monitor (Garmin Forerunner)	Unstable angina, MI, PCI, CABG + low to moderate risk	3 center-based training sessions, followed by a home-based exercise program with training data uploaded to a web application and telephone feedback by a PT 1x/week; 12 weeks	Center-based CR II	Exercise capacity, QOL	Both groups had significantly improved peak VO2, peak workload, and QOL with no difference between groups.
Kraal (2017)	HR monitor (Garmin Forerunner), chest strap	Unstable angina, MI, PCI, CABG + low to moderate risk	3 center-based training sessions, followed by a home-based exercise program with training data uploaded to a web application and telephone feedback by a PT 1x/week; 12 weeks	Center-based CR II	Cost-effectiveness, exercise capacity, PA, psychological status, QOL	Both groups had significantly improved peak VO2 and peak workload with no difference between groups at 12-week follow up and after 1-year follow up. The intervention appeared more cost-effective than the control.
Lear (2015)	HR monitor (Polar)	ACS, PCI, CABG + reside in isolated area + low or moderate risk	Internet-based virtual CR program that included HR monitoring, education, and chat sessions with a nurse, exercise specialist, and dietician; 4 months	Usual care	CV risk factors, exercise capacity	The intervention group had significantly improved maximal time on a treadmill test and dietary quality compared to the control group.
Lee (2013)	ECG monitor (HeartCall)	ACS + PCI	Home-based exercise program with telephone feedback 1x/week + preventative education prior to hospital discharge; N/A	Usual care	Exercise capacity, QOL	Both groups had significantly improved METs; however, the intervention group had significantly greater improvement in METs and improved QOL compared to the control group.
Maddison (2019)	Chest sensor (BioHarness)	Angina, CAD, MI, PCI, CABG diagnosis within 6 months	Home-based exercise program and coaching with behavioral strategies; 12 weeks	Center-based CR II	Costs, CV risk factors, exercise capacity, PA, QOL	Both groups had significantly improved peak VO2 with no difference between groups. The only significant between-group differences were in waist and hip circumferences at week 12 favoring the intervention group. The average cost of center-based and home-based CR were $9,535 and $4,920, respectively.
Marchionni (2003)	HR monitor, wrist	MI	Center-based CR II OR 4–8 center-based training sessions, followed by a home-based exercise program with feedback from home visits by a PT 1x/2 weeks; N/A	Usual care- single education session and routine healthcare utilization	Costs, exercise capacity, QOL	Both CR groups had significantly improved total work capacity but not the control group. QOL improved significantly in middle-aged and old patients regardless of treatment assignment; however, QOL only improved in very old patient who received either CR and not usual care. The average costs of center-based CR, home-based CR, and usual care were $21,298, $13,246, and $12,433, respectively.
Nabutovsky (2022)	Health watch	MI	Home-based exercise program supported by a mobile app that transmits data to a remote care platform; 6 months	None (compared older and younger adults)	Adherence, exercise capacity, patient satisfaction	Participants adhered to the program and most achieved the prespecified goals. Older patients had higher adherence and were more likely to reach their goals compared to younger patients. Older patients had significant improvement in exercise capacity.
Park (2021)	Health watch (Fitbit Charge) vs. pedometer (Walking 3D)	CVD	Home-based exercise program supported by a mobile app to record exercise and receive motivational and educational messages 3x/week; N/A	Recorded daily step count from pedometer	Daily steps, exercise capacity, psychological status, QOL	The intervention group had significantly higher mean daily step count compared to the control group. There were no between-group differences for changes in 6-minute walk distance, depression, or self-efficacy to maintain exercise.
Prince (2018)	Activity monitor (activPAL), thigh	CAD	Home-based exercise program with sedentary prompts from wearable device; 8-weeks	Usual care	CV risk factors, daily steps, exercise capacity, psychological status, QOL, sedentary level	There were no significant between-group differences for changes in peak VO2, daily step counts, or sedentary time. Although, the intervention group had significantly better self-reported physical QOL scores compared to the control group.
Rawstorn (2018)	Wearable sensor	Angina, MI, PCI, CABG within 6 months	Home-based exercise program with real-time physiological monitoring and coaching with behavioral strategies delivered though a platform; 12 weeks	Center-based CR II	Intervention acceptability, demand, and usability	Most participants positively evaluated the usability and acceptability of the intervention. 87% of participants reported that they would choose the intervention if it was available as a usual care service.
Salvi (2018)	Wearable sensor (HR, ECG, RR, workload)	MI, PCI, CABG	Home-based exercise program through a smartphone-guided training system with feedback provided through a dedicated tablet; N/A	Center-based CR II	Intervention acceptability and usability, knowledge level, PA	Participants who completed the intervention positively evaluated the usability and acceptability of the intervention. The intervention group had significantly improved heart-related knowledge compared to the control group.
Scalvini (2009)	ECG monitor (Card-Guard)	CABG, valve replacement	Home-based exercise program with provided ergometer, contact with telemedicine services, and home visits by a nurse or PT at least 1x/week; N/A	None	Exercise capacity, patient satisfaction	Participants had significantly improved 6-minute walk distance. Overall patient satisfaction was about 95%.
Song (2020)	HR monitor (Suunto), waist	CAD by LHC	Home-based exercise program using a software on patient’s smartphones and telephone or text feedback 1x/week; N/A	Usual care	Exercise capacity, PA	The intervention group had significantly improved peak VO2 and exercise habits compared to the control group.
**Patients with heart failure**						
Frederix (2015)	Accelerometer (Yorbody)	HFrEF, HFpEF, PCI, CABG,	Center-based CR II + home-based CR starting at week 6 of 12 of the center-based CR; 24 weeks	Center-based CR II	CV risk factors, exercise capacity, PA, QOL	The intervention group had significantly improved peak VO2, PA, and QOL compared to the control group. The two groups had no difference in CV risk factors outcomes.
Frederix (2016)	Accelerometer (Yorbody)	HFrEF, HFpEF, PCI, CABG,	Center-based CR II + home-based CR starting at week 6 of 12 of the center-based CR; 24 weeks	Center-based CR II	Cost-effectiveness, CV readmission	The intervention group had significantly lower number of days lost due to CV rehospitalizations; higher proportion of actual to theoretical maximal days alive and out of the hospital; and improved cost-effectiveness compared to the control group.
Frederix (2017)	Accelerometer	HFrEF, HFpEF, PCI, CABG,	Center-based CR II + home-based CR starting at week 6 of 12 of the center-based CR; 24 weeks	Center-based CR II	Cost-effectiveness, CV readmission, CV risk factors, exercise capacity, PA, QOL	The intervention group had significantly better peak VO2, PA, QOL, and cost-effectiveness compared to the control group at long term follow up.
Frith (2021)	Accelerometer, wrist	CHF, angina, CAD, MI, CABG, PCI, valve surgery	Home-based CR with the Active+me platform and feedback at least 1x/3 weeks; 8 weeks	None	CV risk factors, daily steps, PA, patient activation, psychological status	Participants had significantly improved patient activation scores, PA, systolic blood pressure, and waist circumference after the intervention. There was no change in daily steps, body mass index, diastolic blood pressure, depression, or anxiety.
Jafri (2022)	Pedometer	CHF, STEMI, NSTEMI, PCI, CABG, valve disease, CAD, PAD	Home-based daily exercises with provided portable peddler and elastic bands and weekly call from CR staff to assess progress and for counseling; 12 weeks	Referred to HBCR but did not attend	All-cause mortality/hospitalization, all-cause mortality, all-cause hospitalization, CV hospitaliztion	The intervention group had significantly lower risk of combined all-cause mortality and all-cause hospitalizations up to 12 months.
Kikuchi (2021)	ECG monitor (Hitoe)	HF + difficulty participating in a center-based CR program	Home-based CR using a provided ergometer with a platform that allowed real-time supervision from a remote medical site; 12 weeks	None	Adherence, CV risk factors, exercise capacity	Median participation rate in the exercise sessions was 94.4%. Six-minute walk test distance significantly improved, but other measures of exercise capacity and CV risk factors did not.
Nagatomi (2022)	Health watch (Fitbit)	Chronic HF + NYHA class II-III, AHA/ACC stage C or D + physical frailty	Technology-based self-management program for exercise and nutrition with feedback from the CR team about 1x/day; 3 months	Usual care	CV risk factors, daily steps, exercise capacity, frailty, QOL	The intervention group had significantly improved 6-minute walk distance and lower extremity muscle strength compared to the control group.
Ngeno (2022)	Pedometer, hip	Chronic HF + NYHA II-III	Home-based daily waking program with telephone feedback by the study coordinator 1x/week; 12 weeks OR center-based CR comprised of 36 tailored exercise sessions	Usual care	Adherence, exercise capacity	Adherence rates in the center-based and home-based CR groups were 46% and 29%, respectively. 6-minute walk distance improved significantly across all three arms.
O’Connor (2009)	HR monitor (Polar)	LVEF ≤35% + NYHA II to IV despite GDMT for ≥6 weeks	36 supervised exercise sessions over 3 months, followed by home-based exercise with provided treadmill or ergometer + usual care; up to 4 years	Usual care- education booklet	All-cause mortality/hospitalization, CV mortality/HF hospitalization, CV mortality/hospitalization	The intervention group had significantly reduced composite CV mortality/HF hospitalization after adjusting for prognostic factors compared to the control group. There was no significant between-group difference for composite all-cause mortality/hospitalization or composite CV mortality/hospitalization.
Peng (2018)	HR monitor	HF diagnosis within 3m + NYHA I to III with GDMT ≥4 weeks	Home-based exercise training using webcam communication and telephone or instant messaging feedback by a cardiac nurse at least 1x/week; 8 weeks	Usual care- discharge education and regular clinic visits	Exercise capacity, LVEF, NYHA classification, psychological status, QOL, resting HR	The intervention group had significantly improved 6-minute walk distance, resting HR, and QOL compared to the control group. There were no between-group differences for anxiety, depression, NYHA classification, or LVEF.
Piotrowicz (2010)	ECG monitor (EHO mini)	Hospitalized + LVEF ≤40% + NYHA II to III despite GDMT ≥4 weeks	Home-based exercise by continuous walking on level ground with health data transmitted to a monitoring center and telephone contact for psychological support; 8 weeks	Center-based ergometer training with PA monitoring	Exercise capacity, NYHA classification, QOL	Both groups had significantly improved NYHA classification, 6-minute walk distance, peak VO2, and QOL. Improvement was greater in the intervention group for NYHA classification and greater in the control group for 6-minute walk distance.
Piotrowicz (2015)	ECG monitor (EHO mini)	LVEF ≤40% + NYHA II to III despite GDMT ≥4 weeks	Home-based exercise by Nordic walking training with health data transmitted to a monitoring center and telephone contact for psychological support; 8 weeks	Usual care- recommendations for lifestyle changes	Exercise capacity, QOL	The intervention group had significantly improved peak VO2, 6-minute walk distance, and QOL compared to the control group.
Piotrowicz (2020)	ECG monitor (EHO mini)	LVEF ≤40% + NYHA I to III + hospitalized within 6 months	5 supervised training sessions, followed by home-based exercise with health data transmitted to a monitoring center; 9 weeks	Usual care for their clinical status- some CR, some remote monitoring of CIEDs	All-cause hospitalization, all-cause mortality, CV hospitalization, CV mortality, exercise capacity, HF hospitalization, NYHA classification, QOL	The intervention group had significantly greater improvement in peak VO2, 6-minute walk distance, QOL, and NYHA classification compared to the control group after 9 weeks. However, there were no between-group differences in percentage of days alive and out of the hospital, mortality, hospitalization, or composite endpoints combining mortality and hospitalization at long term follow up.
Smolis-Bak (2015)	ECG monitor	LVEF ≤35% + NYHA III + CRT-D implantation	CR I, followed by home-based exercise with health data transmitted to a monitoring center; average 11 weeks	CR I with no training after discharge	Exercise capacity, psychological status, QOL	The intervention group had significantly improved peak VO2, treadmill test duration, and METs at 3-4-month follow up, but these values returned to baseline at 12-month follow up. The control group had smaller improvement in these measures at 3-4-month follow up, but the improvement was maintained at 12-month follow up. Depressive symptoms improved in the intervention group at both time points but not the control group.
Tousignant (2019)	ECG monitor	LVEF ≤40% + NYHA I to III	Home-based exercise with health data transmitted to a clinician and decreasing amount of real-time remote supervision; 12 weeks	Usual care- preventative education and encouragement of daily walks	Exercise capacity, QOL	Most participants showed a tendency to improve their exercise capacity. QOL scores improved in all 4 patients.
**Patients with heart valve repair or replacement**						
Brocki (2022)	Health watch (Fitbit Charge or Beurer AS 87)	Post elective transaortic valve implantation (TAVI)	Web-based home exercise training, patient support, the use of an activity tracker, and access to a project website; 3 weeks	None	Daily steps, technical issues	There was large variation in the number of steps take per day from 1868 to 17280. Technical issues were experienced by both patients and healthcare professionals. Telerehabilition for the elderly after TAVI delivered as a web-based intervention does not seem feasible as 60% of the patients did not complete the study.
**Patients with exposure to center-based cardiac rehabilitation**						
Avila (2018)	HR monitor (Garmin Forerunner)	CAD by LHC, MI + completed center-based CR II	3 center-based training sessions, followed by a home-based exercise program with telephone or e-mail feedback 1x/week; 12 weeks OR center-based CR II	Usual care- advice to remain physically active	CV risk factors, exercise capacity, daily steps, PA, QOL, sedentary level	Both CR groups had significantly greater improvement in peak VO2 compared to the control group. The center-based CR group had significantly increased sedentary time over time. There were no significant differences in PA, daily steps, CV risk factors or QOL between groups over time.
Avila (2020)	HR monitor (Garmin Forerunner)	CAD by LHC, MI + completed center-based CR II	3 center-based training sessions, followed by a home-based exercise program with telephone or e-mail feedback 1x/week; 12 weeks OR center-based CR II	Usual care- advice to remain physically active	CV risk factors, exercise capacity, daily steps, PA, QOL, sedentary level	Both CR groups maintained improvements in exercise capacity, PA, and QOL at 1 year follow up.
Butler (2009)	Pedometer (Yamax Digiwalker)	Completed first session of center-based group CR II	Daily step count recording with step goals and telephone feedback at weeks 1, 3, 12, 18; N/A	Usual care- brochures	CV risk factors, exercise capacity, PA, psychological status	The intervention group had significantly improved PA compared to the control group at 6-week and 6-month follow up. The intervention group had significantly improved METs and psychological health.
Cupples (2013)	Pedometer (Yamax Digiwalker)	Completing center-based CR II	Daily step count recording with feedback given by a facilitator face-to-face 1x/week with goal to increase average daily step count by 10% each week to end goal of 10,000 steps/day; N/A	Step count recorded without feedback + face-to-face or telephone contact 1x/week	Daily steps, QOL	The intervention group had significantly increased daily step counts compared to the control group. There was no between-group difference in QOL.
Dolansky (2010)	HR monitor (Polar Vantage), wrist	MI, PCI, CABG + completing center-based CR II	Exercise diary with exercise prescriptions from CR; 12 months	None	Adherence	There was no difference in training adherence between the age groups for women. Older men were non-adherent sooner than younger men.
Duscha (2018)	Health watch (Fitbit Charge)	Completed center-based CR II	Exercise prescription from CR with health coaching; 12 weeks	Usual care- as provided by their physician	Exercise capacity, daily steps, PA	There was a significant between-group difference in peak VO2 and PA at follow up favoring the intervention group. Daily step count trended up in the intervention group and trended down in the control group at follow up.
Guiraud (2012)	Accelerometer, waist	HF, CAD, post-CV surgery + completed center-based CR II + non-compliant with PA	PA recording with telephone feedback by a kinesiologist 1x/15 days; 8 weeks	Step count recorded without feedback	PA	The intervention group that significantly improved weekly time spent doing moderate-intensity PA and weekly active energy expenditure compared to the control group.
Hakala (2021)	Health watch (Fitbit Charge)	Angina, MI, PCI, CABG + attending center-based CR II	Center-based CR II + technology-enabled PA recording with feedback by a PT 1x/month + internet software with exercise instructions and to set/monitor individual goals; 12 months	Center-based CR II + paper-based self-monitoring and exercise instruction	Daily steps, PA	The intervention group had significantly increased weekly time spent doing light-intensity PA at 6-month follow up. However, there were no significant between-group differences in daily step counts or weekly time spent doing light or moderate to vigorous PA at 12-month follow up.
Hannan (2021)	HR monitor (Lynk2)	ACS, PCI, CABG, vascular surgery + completed CR II	PA self-monitoring through a smartphone application with meetings with a PT at weeks 0, 3, 6; 6 weeks	None	PA	PA significantly increased after participants could view their PA data.
Izawa (2005)	Pedometer (Kenz Lifecorder)	MI + completed CR I	Center-based CR II, followed by PA self-monitoring; 6 months + 6 months	Center-based CR II only	Daily steps, exercise capacity, self-efficacy for PA	Both groups had significantly improved peak VO2 with no difference between groups. The intervention group had significantly improved self-efficacy for PA and daily step count compared to the control group.
Saitoh (2022)	Pedometer	Stable cardiac disease + completed CR II	PA self-monitoring through a tablet computer and remote real-time telemedicine system with feedback by a PT 1x/week; 4 weeks	Center-based CR II	Functional status, CV hospitalization	There was no difference in functional status or CV hospitalization between groups.
Skobel (2017)	Wearable sensor, vest	MI, PCI, CABG + completed center-based CR II	PA self-monitoring through a smartphone-guided training system with continuous review by a dedicated team; 6 months	Daily PA recorded on a paper diary	CV risk factors, exercise capacity, LVEF, QOL	The intervention group had significantly improved peak VO2 compared to the control group. LVEF increased in the intervention group but significantly decreased in the control group. There was no between-group differences for CV risk factors or QOL.
Snoek (2021)	HR monitor (Zephyr), waist	ACS, PCI, CABG within 3m of CR II + completed CR II	PA self-monitoring through an individual webpage and on their smartphone with telephone feedback 1x/week to 1x/month + usual care; 6 months	Usual care- seen by their cardiologist 3 and 12 months post-CR II	CV risk factors, exercise capacity, PA, psychological status, QOL	Both groups had significantly improved peak VO2 and peak workload with no difference between groups. There were no significant changes or between-group differences for QOL, CV risk factors, PA, or psychological status.
Vieira (2017)	Accelerometer (ActiGraph)	CAD, stable angina, MI + completed CR II	Home-based CR III using a computer and Microsoft Kinect (virtual reality) OR home-based CR III using a paper booklet; N/A	Usual care- CV risk factor education	CV risk factors, PA	The home-based virtual reality CR III group had significantly improved waist-to-hip ratio compared to the conventional CR III or usual care group at 3- and 6-month follow up.

Abbreviations: ACS acute coronary syndrome; BP blood pressure; CABG coronary artery bypass graft; CIED cardiovascular implantable electronic devices; CM cardiomyopathy; CR cardiac rehabilitation; CV cardiovascular; ECG electrocardiogram; ER emergency room; HR heart rate; LHC left heart catheterization; LVEF left ventricular ejection fraction; METs metabolic equivalent of the tasks; MI myocardial infarction; NSTEMI non-ST elevation myocardial infarction; NYHA New York Heart Association; PA physical activity; PCI percutaneous coronary intervention; PT physical therapy or physical therapist; QOL quality of life; STEMI ST elevation myocardial infarction; UA unstable angina.

CV risk factors include anthropometric measurements (i.e. BMI, waist circumference), laboratory tests (i.e. lipid profile, HbA1c), BP, smoking status, and diet.

PA include exercise habits, PA energy expenditure, and PA level (i.e. daily sedentary, light, moderate, and vigorous activity duration).

Psychological scores included those for depression, anxiety, and self-efficacy.

### Patients with CHD (without HF)

Most studies were conducted among patients with obstructive coronary artery disease, but 4 studies did not specifically include these patients [18, 27, 47, 68]. Out of the 27 studies in this group, 21 measured exercise capacity, 15 measured QOL, 8 measured PA/sedentary level or CV risk factors, 6 measured psychological status, 5 measured daily step count, 4 measured user acceptability/satisfaction, 2 measured cost/cost-effectiveness, and 1 measured rehospitalization.

Five studies noted improvement in peak VO2 in both groups [12, 29, 38, 39, 58], while 5 studies noted significant improvement in the intervention group [21, 31, 47, 54, 55], however, 2 studies noted no difference in peak VO2 between intervention and control group [27, 30]. Five studies noted QOL improvement in both groups [12, 29, 37, 38, 56], 3 studies noted QOL improvement in intervention group [45, 64, 68], while only one study noted QOL improvement in control group [48]. One study noted no change in QOL [14]. Intervention groups in 6 studies had a significant increase in daily steps from baseline and improvement in walking time and exercise habits [18, 30, 45, 57, 68, 69], while one study noted no significant change in daily steps [27]. Two studies found that HBCR was cost effective compared to center-based CR [56, 58]. In one study, 87% of patients reported that they would choose the intervention if available as part of usual care [59].

### Patients with HF

Nine studies were conducted in patients with HF of any left ventricular ejection fraction (LVEF) [19, 21–23, 34, 35, 46, 63, 66], while six studies included patients with heart failure with reduced ejection fraction (LVEF ≤40%) only [17, 28, 41–44]. Nine studies had New York Heart Association (NYHA) classification criteria [17, 28, 35, 41–44, 46, 63], while 6 studies did not [19, 21–23, 34, 66].

Out of the 15 studies in this group, 11 measured exercise capacity; 9 measured QOL; 5 measured CV risk factors and/or hospitalization; 3 measured mortality, NYHA classification, PA, and/or psychological status; 2 measured adherence, cost-effectiveness, and/or daily step count; and 1 measured frailty, LVEF, patient activation, and/or resting heart rate.

Six studies noted significant improvement in peak VO2 in intervention group [21, 23, 41–44], and 6 studies noted significant improvement in QOL [21, 23, 28, 41–43, 46]. Two studies noted significant improvement in PA in the intervention group compared to the control [21, 23]. There was also a significant cost effectiveness in the intervention group compared to control group [21, 23]. Two studies noted significant reduction in composite CV mortality and heart failure hospitalization [17, 19], whereas another study noted no significant difference in either group [42].

### Patients with exposure to center-based CR

Majority of these studies were conducted in patients with coronary heart disease and only 1 study included patients with heart failure [53]. All of the patients in these studies had exposure to center-based CR prior to starting HBCR. Out of the 14 studies in this group, 10 measured PA, 6 measured exercise capacity, 5 measured CV risk factors and/or daily step count, 4 measured QOL, 2 measured psychological status, and 1 measured LVEF, sedentary level, self-efficacy for PA, functional status, or training adherence.

Three studies noted improved peak VO2 in intervention group [16, 20, 50], while one study noted peak VO2 improvement in both groups [40]. Four studies noted no improvement in QOL in either group [20, 40, 50, 67]. Five studies noted significant PA improvement in the intervention group at the end of the intervention [16, 33, 51, 53, 61], while 2 studies noted no significant difference in the control or intervention group. In addition, 3 studies noted PA improvement at follow up [16, 25, 51]; however, one study noted no PA improvement at follow up in the control or intervention group [61]. The intervention group had a significant improvement or uptrend in daily step count [16, 33, 67], while there was no improvement in 2 studies [20, 61]. One study noted the intervention group had significant improvement in waist to hip ratio, compared to the center-based CR group that had an increase in sedentary time [65]. LVEF significantly improved in the intervention group, while no improvement or a decrease in LVEF was noted in the control group [50]. One study also noted there was no difference in adherence to training between different age groups of women compared to men [15].

### Patients with heart valve repair or replacement

One study explored HBCR in patients after transaortic valve implantation (TAVI). Most patients were elderly (>80 years old) and 60% were men. The small cohort consistent of thirteen patients underwent HBCR with two different devices. By the end of the study, there was a large variability of physical activity between participants and a significant drop-out rate of 60%. Both physicians and patients faced many technical challenges during the intervention period. In this group of patients, HBCR was not feasible [58].

Overall, in three of the main study groups, there was a trend toward improvement in QOL and peak VO2, less sedentary time, and an increase in daily step count in the intervention groups compared to control groups.

## Discussion

In this scoping review, the goal was to better understand the role wearable devices play in HBCR and to characterize the ways in which wearable devices have been used. In reviewing the articles that met the inclusion criteria, we found four main categories of studies: patients enrolled in HBCR for CHD (without HF); patients enrolled in HBCR for HF; patients who also had exposure to center-based CR; and patients enrolled in HBCR for heart valve repair or replacement. Except for patients with heart valve repair or replacement, each category had a substantial number of studies that met that criterion, allowing us to look at wearable devices in HBCR from both a more general and focused view.

Our study shows that there are many different wearable devices that can be utilized in CR. The most commonly used devices were heart rate monitors followed by ECG monitors and accelerometers. Pedometers and health watches were used slightly less frequently, and six studies [27, 45, 49, 50, 58, 59] mentioned another type of device. Regardless of the device used, HBCR seems to be an effective alternative to center-based CR. Despite the variable cost between devices, most studies showed they could be effectively used in HBCR [15, 52].

Of the 57 studies reviewed, only one had equal male and female representation [55]. Although CVD continues to be a leading cause of death for women, the CR referral rates for women are significantly lower than men [70]. Healthcare disparities between genders continues to be a significant obstacle for RCTs, and this review continues to emphasize this point. Therefore, statements about HBCR can only be generalized to men. More research regarding HBCR for women must be conducted.

Patients often express that adherence to CR is difficult because of barriers such as cost and lack of flexibility in scheduling [8, 9]. As such, HBCR is a desirable option to improve compliance. Our study demonstrates that HBCR is comparable to center-based CR in three patient groups. This is further supported by 44 of the 57 studies being RCTs. RCTs reduce selection bias of patients who might be more motivated to complete HBCR; thus, this further supports HBCR being a comparable option.

The duration of many of the studies in our review were less than three months. Only two studies reported 1 year follow-up data of previously published studies [25, 32]. Thus, more work should be done to follow patients over a longer period to see the long-term benefits and drawbacks of HBCR compared to center-based CR. With the current data, it is hard to make conclusions about the long-term sustainability of HBCR.

Additionally, a key component of CR is risk factor management and education [1]. In the studies that discussed CV risk factors, seven studies reported that there was no difference between the two groups. This shows that HBCR is as effective at managing risk factors as center-based CR further making the case that HBCR is a comparable option.

### Patients with CHD (without HF)

Many of the studies included in this group compared HBCR to center-based CR. Center-based CR has proven to be effective at reducing cardiovascular risk factors, improving overall patient wellbeing, and QOL [1]. It is imperative that home-based interventions meet the same standards. As more studies are done regarding this topic, there is growing evidence in support of home-based methods as appropriate alternatives, providing patients with more flexibility and less expenses [1]. The studies that we reviewed correlated with these findings, as many studies found no differences between the two groups with both groups showing improvement in the respective measured outcomes. Of note, a few studies noted that the intervention group had a greater improvement in QOL than the control group [27, 45, 64, 68]. Thus, one may argue that this is a result of the flexibility of home-based interventions and their ability to fit more compatibly into one’s daily routine.

### Patients with HF

Similarly to the CHD category, many of the studies in this subset compared home-based exercise interventions with usual care or centered-based CR and showed an increase in QOL, exercise capacity, and peak VO_2_ in the intervention group compared to the control group. Of note, two studies in this group focused on cost effectiveness [22, 23], and 5 studies focused CV readmission or CV hospitalization [17, 19, 22, 23, 42]. These outcomes were not as prevalently studied in the articles included in the other categories. Cost effectiveness was shown to be better in the intervention groups [22, 23], again making the case for expanding HBCR from an economic perspective. Furthering this economic argument, patients in the intervention groups also had decreased hospitalizations compared to the control groups [17, 22]. Thus, HBCR can be an effective way in saving healthcare costs.

### Patients with exposure to center-based CR

In this subset of studies, most interventions involved PA monitoring after center-based CR. They then compared patients who were instructed to remotely monitor their PA with those who were in the usual care group. A majority of studies found that the intervention group had increased PA over time. It is important for patients to maintain the improvements that they make in center-based CR, and these studies make a case for the use of wearable devices for PA monitoring. If patients know that their data is being recorded, they may be more likely to continue their exercise regimen which leads to more long-term benefits.

### Patients with heart valve repair or replacement

The only RCT exploring HBCR in post-TAVI patients had a very small cohort in an elderly population (>80 years old). Due to technical issues, there was a large percentage of the cohort who did not complete the study. As TAVI is becoming increasingly popular as one of the standards of care for severe aortic stenosis, more RCTs are required in this population to determine a real benefit for HBCR.

### Limitations

As technology continues to advance, there are more and better wearable devices on the market that can be utilized during CR. This is an area of active research, and our review is a snapshot of the studies at the time that the search was conducted in March 2023. Additionally, the COVID-19 pandemic significantly changed how physicians and healthcare systems think about telemedicine and telerehabilitation. Although our search was conducted in early 2023, it may not reflect the full effect of the pandemic on how physicians approach CR. Lastly, because most of the patients evaluated in the included studies were males, there might be a lack of generalizability reflected in our results.

## Conclusion

This review included 57 articles discussing the role of wearable devices in HBCR. These studies were divided into four main categories: patients with CHD without HF, patients with HF, patients with exposure to center-based CR, and patients with heart valve repair or replacement. Our study shows that wearable devices and HBCR can be a comparable alternative or adjunct to center-based CR for patients with CHD and HF. When comparing center-based CR and HBCR, HBCR leads to an improved QOL and peak VO2, less sedentary time, and an increase in daily step count. It is also more cost effective. More studies are needed to draw conclusions about the comparability of HBCR to center-based CR in patients with heart valve repair or replacement.

## Supporting information

S1 Checklist Preferred Reporting Items for Systematic reviews and Meta-Analyses extension for Scoping Reviews (PRISMA-ScR) checklist. (PDF)

## References

[1] Taylor RS, Dalal HM, McDonagh STJ. The role of cardiac rehabilitation in improving cardiovascular outcomes. Nat Rev Cardiol. 2022;19(3):180–94. doi: 10.1038/s41569-021-00611-7 34531576 PMC8445013

[2] Thomas RJ, Balady G, Banka G, Beckie TM, Chiu J, Gokak S, et al. 2018 ACC/AHA Clinical Performance and Quality Measures for Cardiac Rehabilitation: A Report of the American College of Cardiology/American Heart Association Task Force on Performance Measures. J Am Coll Cardiol. 2018;71(16):1814–37. 29606402 10.1016/j.jacc.2018.01.004

[3] Thomas RJ, Beatty AL, Beckie TM, Brewer LC, Brown TM, Forman DE, et al. Home-Based Cardiac Rehabilitation: A Scientific Statement From the American Association of Cardiovascular and Pulmonary Rehabilitation, the American Heart Association, and the American College of Cardiology. J Am Coll Cardiol. 2019;74(1):133–53. doi: 10.1016/j.jacc.2019.03.008 31097258 PMC7341112

[4] CQMC Core Sets: Core Quality Measures Collaborative; Available from: https://www.qualityforum.org/cqmc/.

[5] Ometov A, Shubina V, Klus L, Skibińska J, Saafi S, Pascacio P, et al. A survey on wearable technology: History, state-of-the-art and current challenges. Computer Networks. 2021;193:108074.

[6] Sparaco M, Lavorgna L, Conforti R, Tedeschi G, Bonavita S. The Role of Wearable Devices in Multiple Sclerosis. Mult Scler Int. 2018;2018:7627643. doi: 10.1155/2018/7627643 30405913 PMC6199873

[7] Abbadessa G, Lavorgna L, Miele G, Mignone A, Signoriello E, Lus G, et al. Assessment of Multiple Sclerosis Disability Progression Using a Wearable Biosensor: A Pilot Study. J Clin Med. 2021;10(6). doi: 10.3390/jcm10061160 33802029 PMC8001885

[8] Bayoumy K, Gaber M, Elshafeey A, Mhaimeed O, Dineen EH, Marvel FA, et al. Smart wearable devices in cardiovascular care: where we are and how to move forward. Nature Reviews Cardiology. 2021;18(8):581–99. doi: 10.1038/s41569-021-00522-7 33664502 PMC7931503

[9] McMahon SR, Ades PA, Thompson PD. The role of cardiac rehabilitation in patients with heart disease. Trends Cardiovasc Med. 2017;27(6):420–5. doi: 10.1016/j.tcm.2017.02.005 28318815 PMC5643011

[10] Sana F, Isselbacher EM, Singh JP, Heist EK, Pathik B, Armoundas AA. Wearable Devices for Ambulatory Cardiac Monitoring: JACC State-of-the-Art Review. J Am Coll Cardiol. 2020;75(13):1582–92. 32241375 10.1016/j.jacc.2020.01.046 PMC7316129

[11] Page MJ, McKenzie JE, Bossuyt PM, Boutron I, Hoffmann TC, Mulrow CD, et al. The PRISMA 2020 statement: an updated guideline for reporting systematic reviews. Bmj. 2021;372:n71. doi: 10.1136/bmj.n71 33782057 PMC8005924

[12] Ades PA, Pashkow FJ, Fletcher G, Pina IL, Zohman LR, Nestor JR. A controlled trial of cardiac rehabilitation in the home setting using electrocardiographic and voice transtelephonic monitoring. Am Heart J. 2000;139(3):543–8. doi: 10.1016/s0002-8703(00)90100-5 10689271

[13] Chokshi NP, Adusumalli S, Small DS, Morris A, Feingold J, Ha YP, et al. Loss-Framed Financial Incentives and Personalized Goal-Setting to Increase Physical Activity Among Ischemic Heart Disease Patients Using Wearable Devices: The ACTIVE REWARD Randomized Trial. J Am Heart Assoc. 2018;7(12). doi: 10.1161/JAHA.118.009173 29899015 PMC6220554

[14] Ding EY, Erskine N, Stut W, McManus DD, Peterson A, Wang Z, et al. MI-PACE Home-Based Cardiac Telerehabilitation Program for Heart Attack Survivors: Usability Study. JMIR Hum Factors. 2021;8(3):e18130. doi: 10.2196/18130 34255660 PMC8299347

[15] Dolansky MA, Stepanczuk B, Charvat JM, Moore SM. Women’s and men’s exercise adherence after a cardiac event: does age make a difference? Research in Gerontological Nursing. 2010;3(1):30–8. 20128541 10.3928/19404921-20090706-03 PMC2897096

[16] Duscha BD, Piner LW, Patel MP, Craig KP, Brady M, McGarrah RW, 3rd, et al. Effects of a 12-week mHealth program on peak VO(2) and physical activity patterns after completing cardiac rehabilitation: A randomized controlled trial. Am Heart J. 2018;199:105–14. doi: 10.1016/j.ahj.2018.02.001 29754647

[17] O’Connor CM, Whellan DJ, Lee KL, Keteyian SJ, Cooper LS, Ellis SJ, et al. Efficacy and safety of exercise training in patients with chronic heart failure: HF-ACTION randomized controlled trial. Jama. 2009;301(14):1439–50. doi: 10.1001/jama.2009.454 19351941 PMC2916661

[18] Park LG, Elnaggar A, Lee SJ, Merek S, Hoffmann TJ, Von Oppenfeld J, et al. Mobile Health Intervention Promoting Physical Activity in Adults Post Cardiac Rehabilitation: Pilot Randomized Controlled Trial. JMIR Form Res. 2021;5(4):e20468. doi: 10.2196/20468 33861204 PMC8087971

[19] Jafri SH, Imran TF, Medbury E, Ursillo J, Ahmad K, Imran H, et al. Cardiovascular Outcomes of Patients Referred to Home Based Cardiac Rehabilitation. Heart Lung. 2022;52:1–7. doi: 10.1016/j.hrtlng.2021.11.005 34801771 PMC8600943

[20] Avila A, Claes J, Goetschalckx K, Buys R, Azzawi M, Vanhees L, et al. Home-Based Rehabilitation With Telemonitoring Guidance for Patients With Coronary Artery Disease (Short-Term Results of the TRiCH Study): Randomized Controlled Trial. J Med Internet Res. 2018;20(6):e225. doi: 10.2196/jmir.9943 29934286 PMC6035351

[21] Frederix I, Hansen D, Coninx K, Vandervoort P, Vandijck D, Hens N, et al. Medium-Term Effectiveness of a Comprehensive Internet-Based and Patient-Specific Telerehabilitation Program With Text Messaging Support for Cardiac Patients: Randomized Controlled Trial. J Med Internet Res. 2015;17(7):e185. doi: 10.2196/jmir.4799 26206311 PMC4528085

[22] Frederix I, Hansen D, Coninx K, Vandervoort P, Vandijck D, Hens N, et al. Effect of comprehensive cardiac telerehabilitation on one-year cardiovascular rehospitalization rate, medical costs and quality of life: A cost-effectiveness analysis. Eur J Prev Cardiol. 2016;23(7):674–82. doi: 10.1177/2047487315602257 26289723

[23] Frederix I, Solmi F, Piepoli MF, Dendale P. Cardiac telerehabilitation: A novel cost-efficient care delivery strategy that can induce long-term health benefits. Eur J Prev Cardiol. 2017;24(16):1708–17. doi: 10.1177/2047487317732274 28925749

[24] Frederix I, Van Driessche N, Hansen D, Berger J, Bonne K, Alders T, et al. Increasing the medium-term clinical benefits of hospital-based cardiac rehabilitation by physical activity telemonitoring in coronary artery disease patients. Eur J Prev Cardiol. 2015;22(2):150–8. doi: 10.1177/2047487313514018 24249840

[25] Avila A, Claes J, Buys R, Azzawi M, Vanhees L, Cornelissen V. Home-based exercise with telemonitoring guidance in patients with coronary artery disease: Does it improve long-term physical fitness? Eur J Prev Cardiol. 2020;27(4):367–77. doi: 10.1177/2047487319892201 31787026

[26] Lear SA, Singer J, Banner-Lukaris D, Horvat D, Park JE, Bates J, et al. Improving access to cardiac rehabilitation using the internet: a randomized trial. Stud Health Technol Inform. 2015;209:58–66. 25980706

[27] Prince SA, Reed JL, Cotie LM, Harris J, Pipe AL, Reid RD. Results of the Sedentary Intervention Trial in Cardiac Rehabilitation (SIT-CR Study): A pilot randomized controlled trial. Int J Cardiol. 2018;269:317–24. doi: 10.1016/j.ijcard.2018.07.082 30072156

[28] Tousignant M, Mampuya WM, Bissonnette J, Guillemette E, Lauriault F, Lavoie J, et al. Telerehabilitation with live-feed biomedical sensor signals for patients with heart failure: a pilot study. Cardiovasc Diagn Ther. 2019;9(4):319–27. doi: 10.21037/cdt.2019.03.05 31555536 PMC6732086

[29] Batalik L, Dosbaba F, Hartman M, Batalikova K, Spinar J. Benefits and effectiveness of using a wrist heart rate monitor as a telerehabilitation device in cardiac patients: A randomized controlled trial. Medicine (Baltimore). 2020;99(11):e19556. doi: 10.1097/MD.0000000000019556 32176113 PMC7440288

[30] Batalik L, Konecny V, Dosbaba F, Vlazna D, Brat K. Cardiac Rehabilitation Based on the Walking Test and Telerehabilitation Improved Cardiorespiratory Fitness in People Diagnosed with Coronary Heart Disease during the COVID-19 Pandemic. Int J Environ Res Public Health. 2021;18(5). doi: 10.3390/ijerph18052241 33668304 PMC7956401

[31] Batalik L, Pepera G, Papathanasiou J, Rutkowski S, Líška D, Batalikova K, et al. Is the Training Intensity in Phase Two Cardiovascular Rehabilitation Different in Telehealth versus Outpatient Rehabilitation? J Clin Med. 2021;10(18). doi: 10.3390/jcm10184069 34575185 PMC8466823

[32] Batalik L, Dosbaba F, Hartman M, Konecny V, Batalikova K, Spinar J. Long-term exercise effects after cardiac telerehabilitation in patients with coronary artery disease: 1-year follow-up results of the randomized study. Eur J Phys Rehabil Med. 2021;57(5):807–14. doi: 10.23736/S1973-9087.21.06653-3 33619944

[33] Izawa KP, Watanabe S, Omiya K, Hirano Y, Oka K, Osada N, et al. Effect of the self-monitoring approach on exercise maintenance during cardiac rehabilitation: a randomized, controlled trial. Am J Phys Med Rehabil. 2005;84(5):313–21. doi: 10.1097/01.phm.0000156901.95289.09 15829777

[34] Kikuchi A, Taniguchi T, Nakamoto K, Sera F, Ohtani T, Yamada T, et al. Feasibility of home-based cardiac rehabilitation using an integrated telerehabilitation platform in elderly patients with heart failure: A pilot study. J Cardiol. 2021;78(1):66–71. doi: 10.1016/j.jjcc.2021.01.010 33579602

[35] Nagatomi Y, Ide T, Higuchi T, Nezu T, Fujino T, Tohyama T, et al. Home-based cardiac rehabilitation using information and communication technology for heart failure patients with frailty. ESC Heart Fail. 2022;9(4):2407–18. doi: 10.1002/ehf2.13934 35534907 PMC9288767

[36] Saitoh M, Takahashi T, Morisawa T, Honzawa A, Yokoyama M, Abulimiti A, et al. Remote Cardiac Rehabilitation in Older Cardiac Disease: A Randomized Case Series Feasibility Study. Cardiol Res. 2022;13(1):57–64. doi: 10.14740/cr1346 35211224 PMC8827239

[37] Brouwers RWM, Kraal JJ, Regis M, Spee RF, Kemps HMC. Effectiveness of Cardiac Telerehabilitation With Relapse Prevention: SmartCare-CAD Randomized Controlled Trial. Journal of the American College of Cardiology. 2021;77(21):2754–6. doi: 10.1016/j.jacc.2021.03.328 34045031

[38] Kraal JJ, Peek N, Van den Akker-Van Marle ME, Kemps HM. Effects of home-based training with telemonitoring guidance in low to moderate risk patients entering cardiac rehabilitation: short-term results of the FIT@Home study. Eur J Prev Cardiol. 2014;21(2 Suppl):26–31. doi: 10.1177/2047487314552606 25354951

[39] Kraal JJ, Van den Akker-Van Marle ME, Abu-Hanna A, Stut W, Peek N, Kemps HM. Clinical and cost-effectiveness of home-based cardiac rehabilitation compared to conventional, centre-based cardiac rehabilitation: Results of the FIT@Home study. Eur J Prev Cardiol. 2017;24(12):1260–73. doi: 10.1177/2047487317710803 28534417 PMC5518918

[40] Snoek JA, Meindersma EP, Prins LF, Van’t Hof AW, de Boer MJ, Hopman MT, et al. The sustained effects of extending cardiac rehabilitation with a six-month telemonitoring and telecoaching programme on fitness, quality of life, cardiovascular risk factors and care utilisation in CAD patients: The TeleCaRe study. J Telemed Telecare. 2021;27(8):473–83. doi: 10.1177/1357633X19885793 31760855

[41] Piotrowicz E, Baranowski R, Bilinska M, Stepnowska M, Piotrowska M, Wójcik A, et al. A new model of home-based telemonitored cardiac rehabilitation in patients with heart failure: effectiveness, quality of life, and adherence. Eur J Heart Fail. 2010;12(2):164–71. doi: 10.1093/eurjhf/hfp181 20042423

[42] Piotrowicz E, Pencina MJ, Opolski G, Zareba W, Banach M, Kowalik I, et al. Effects of a 9-Week Hybrid Comprehensive Telerehabilitation Program on Long-term Outcomes in Patients With Heart Failure: The Telerehabilitation in Heart Failure Patients (TELEREH-HF) Randomized Clinical Trial. JAMA Cardiol. 2020;5(3):300–8. doi: 10.1001/jamacardio.2019.5006 31734701 PMC6865325

[43] Piotrowicz E, Zieliński T, Bodalski R, Rywik T, Dobraszkiewicz-Wasilewska B, Sobieszczańska-Małek M, et al. Home-based telemonitored Nordic walking training is well accepted, safe, effective and has high adherence among heart failure patients, including those with cardiovascular implantable electronic devices: a randomised controlled study. Eur J Prev Cardiol. 2015;22(11):1368–77. doi: 10.1177/2047487314551537 25261268

[44] Smolis-Bąk E, Dąbrowski R, Piotrowicz E, Chwyczko T, Dobraszkiewicz-Wasilewska B, Kowalik I, et al. Hospital-based and telemonitoring guided home-based training programs: effects on exercise tolerance and quality of life in patients with heart failure (NYHA class III) and cardiac resynchronization therapy. A randomized, prospective observation. Int J Cardiol. 2015;199:442–7. doi: 10.1016/j.ijcard.2015.07.041 26276068

[45] Fang J, Huang B, Xu D, Li J, Au WW. Innovative Application of a Home-Based and Remote Sensing Cardiac Rehabilitation Protocol in Chinese Patients After Percutaneous Coronary Intervention. Telemed J E Health. 2019;25(4):288–93. doi: 10.1089/tmj.2018.0064 30192210

[46] Peng X, Su Y, Hu Z, Sun X, Li X, Dolansky MA, et al. Home-based telehealth exercise training program in Chinese patients with heart failure: A randomized controlled trial. Medicine (Baltimore). 2018;97(35):e12069. doi: 10.1097/MD.0000000000012069 30170422 PMC6392598

[47] Song Y, Ren C, Liu P, Tao L, Zhao W, Gao W. Effect of Smartphone-Based Telemonitored Exercise Rehabilitation among Patients with Coronary Heart Disease. J Cardiovasc Transl Res. 2020;13(4):659–67. doi: 10.1007/s12265-019-09938-6 31820334 PMC7423855

[48] Bravo-Escobar R, González-Represas A, Gómez-González AM, Montiel-Trujillo A, Aguilar-Jimenez R, Carrasco-Ruíz R, et al. Effectiveness and safety of a home-based cardiac rehabilitation programme of mixed surveillance in patients with ischemic heart disease at moderate cardiovascular risk: A randomised, controlled clinical trial. BMC Cardiovasc Disord. 2017;17(1):66. doi: 10.1186/s12872-017-0499-0 28219338 PMC5319164

[49] Salvi D, Ottaviano M, Muuraiskangas S, Martínez-Romero A, Vera-Muñoz C, Triantafyllidis A, et al. An m-Health system for education and motivation in cardiac rehabilitation: the experience of HeartCycle guided exercise. J Telemed Telecare. 2018;24(4):303–16. doi: 10.1177/1357633X17697501 28350282

[50] Skobel E, Knackstedt C, Martinez-Romero A, Salvi D, Vera-Munoz C, Napp A, et al. Internet-based training of coronary artery patients: the Heart Cycle Trial. Heart Vessels. 2017;32(4):408–18. doi: 10.1007/s00380-016-0897-8 27730298

[51] Butler L, Furber S, Phongsavan P, Mark A, Bauman A. Effects of a pedometer-based intervention on physical activity levels after cardiac rehabilitation: a randomized controlled trial. J Cardiopulm Rehabil Prev. 2009;29(2):105–14. doi: 10.1097/HCR.0b013e31819a01ff 19305235

[52] Hannan AL, Hing W, Coombes JS, Gough S, Climstein M, Adsett G, et al. Effect of personal activity intelligence (PAI) monitoring in the maintenance phase of cardiac rehabilitation: a mixed methods evaluation. BMC Sports Sci Med Rehabil. 2021;13(1):124. doi: 10.1186/s13102-021-00350-9 34629086 PMC8503999

[53] Guiraud T, Granger R, Gremeaux V, Bousquet M, Richard L, Soukarié L, et al. Telephone support oriented by accelerometric measurements enhances adherence to physical activity recommendations in noncompliant patients after a cardiac rehabilitation program. Arch Phys Med Rehabil. 2012;93(12):2141–7. doi: 10.1016/j.apmr.2012.06.027 22813832

[54] Dehghani M, Namdari M, Rafieian-Kopaei M, Baharvand-Ahmadi B, Mokhayeri Y, Namdari P, et al. Comparison of the effects of the time of home-based cardiac rehabilitation program on the changes in cardiometabolic risk factors in patients with phase-IV myocardial infarction: A randomized controlled trial. ARYA Atheroscler. 2022;18(1):1–9. doi: 10.48305/arya.v18i0.2167 36818148 PMC9931602

[55] Dehghani M, Cheraghi M, Namdari M, Roshan VD. Effects of Phase IV Pedometer Feedback Home-Based Cardiac Rehabilitation on Cardiovascular Functional Capacity in Patients With Myocardial Infarction: A Randomized Controlled Trial. Int J Basic Sci Med. 2019;4(2):75–80.

[56] Marchionni N, Fattirolli F, Fumagalli S, Oldridge N, Del Lungo F, Morosi L, et al. Improved exercise tolerance and quality of life with cardiac rehabilitation of older patients after myocardial infarction: results of a randomized, controlled trial. Circulation. 2003;107(17):2201–6. doi: 10.1161/01.CIR.0000066322.21016.4A 12707240

[57] Scalvini S, Zanelli E, Comini L, Tomba MD, Troise G, Giordano A. Home-based exercise rehabilitation with telemedicine following cardiac surgery. J Telemed Telecare. 2009;15(6):297–301. doi: 10.1258/jtt.2009.090208 19720767

[58] Maddison R, Rawstorn JC, Stewart RAH, Benatar J, Whittaker R, Rolleston A, et al. Effects and costs of real-time cardiac telerehabilitation: randomised controlled non-inferiority trial. Heart. 2019;105(2):122–9. doi: 10.1136/heartjnl-2018-313189 30150328 PMC6352408

[59] Rawstorn JC, Gant N, Rolleston A, Whittaker R, Stewart R, Benatar J, et al. End Users Want Alternative Intervention Delivery Models: Usability and Acceptability of the REMOTE-CR Exercise-Based Cardiac Telerehabilitation Program. Arch Phys Med Rehabil. 2018;99(11):2373–7. doi: 10.1016/j.apmr.2018.06.027 30076800

[60] Brocki BC, Andreasen JJ, Aaroe J, Andreasen J, Thorup CB. Exercise-Based Real-time Telerehabilitation for Older Adult Patients Recently Discharged After Transcatheter Aortic Valve Implantation: Mixed Methods Feasibility Study. JMIR Rehabil Assist Technol. 2022;9(2):e34819. doi: 10.2196/34819 35471263 PMC9092235

[61] Hakala S, Kivistö H, Paajanen T, Kankainen A, Anttila MR, Heinonen A, et al. Effectiveness of Distance Technology in Promoting Physical Activity in Cardiovascular Disease Rehabilitation: Cluster Randomized Controlled Trial, A Pilot Study. JMIR Rehabil Assist Technol. 2021;8(2):e20299. doi: 10.2196/20299 34142970 PMC8277324

[62] Nabutovsky I, Breitner D, Heller A, Scheinowitz M, Klempfner Y, Klempfner R. The First National Program of Remote Cardiac Rehabilitation in Israel-Goal Achievements, Adherence, and Responsiveness in Older Adult Patients: Retrospective Analysis. JMIR Cardio. 2022;6(2):e36947. doi: 10.2196/36947 36383410 PMC9713616

[63] Ngeno GTK, Barasa F, Kamano J, Kwobah E, Wambui C, Binanay C, et al. Feasibility of Cardiac Rehabilitation Models in Kenya. Ann Glob Health. 2022;88(1):7. doi: 10.5334/aogh.3392 35087707 PMC8782083

[64] Lee YH, Hur SH, Sohn J, Lee HM, Park NH, Cho YK, et al. Impact of home-based exercise training with wireless monitoring on patients with acute coronary syndrome undergoing percutaneous coronary intervention. J Korean Med Sci. 2013;28(4):564–8. doi: 10.3346/jkms.2013.28.4.564 23580444 PMC3617309

[65] Vieira ÁSdS, Cristina Damas Argel de Melo M, Andreia Raquel Santos Noites SP, Machado JP, Joaquim Gabriel MM. The effect of virtual reality on a home-based cardiac rehabilitation program on body composition, lipid profile and eating patterns: A randomized controlled trial. European Journal of Integrative Medicine. 2017;9:69–78.

[66] Frith G, Carver K, Curry S, Darby A, Sydes A, Symonds S, et al. Changes in patient activation following cardiac rehabilitation using the Active(+)me digital healthcare platform during the COVID-19 pandemic: a cohort evaluation. BMC Health Serv Res. 2021;21(1):1363. doi: 10.1186/s12913-021-07363-7 34952575 PMC8703006

[67] Cupples M, Dean A, Tully MA, Taggart M, McCorkell G, O’Neill S, et al. Using pedometer step-count goals to promote physical activity in cardiac rehabilitation: a feasibility study of a controlled trial. Int J Phys Med Rehabil. 2013;1(7):1–5.

[68] Devi R, Powell J, Singh S. A web-based program improves physical activity outcomes in a primary care angina population: randomized controlled trial. J Med Internet Res. 2014;16(9):e186. doi: 10.2196/jmir.3340 25217464 PMC4180351

[69] Chokshi N, Adusumalli S, Small D, Morris A, Feingold J, Ha Y, et al. Effect of loss-framed financial incentives and personalized goal-setting on physical activity among ischemic heart disease patients using wearable devices: The active reward randomized clinical trial. Journal of General Internal Medicine. 2018;33(2):176. 10.1161/JAHA.118.009173 PMC6220554 29899015

[70] Colella TJ, Gravely S, Marzolini S, Grace SL, Francis JA, Oh P, et al. Sex bias in referral of women to outpatient cardiac rehabilitation? A meta-analysis. Eur J Prev Cardiol. 2015;22(4):423–41. doi: 10.1177/2047487314520783 24474091

